# Facile Iodine-Catalyzed Michael Addition of Indoles to **α**,**α**′-Bis(arylmethylene)cyclopentanones: An Efficient Synthesis of *E*-2-(3-Indolylphenylmethyl)-5-phenylmethylenecyclopentanones

**DOI:** 10.5402/2012/674629

**Published:** 2012-11-05

**Authors:** Rammohan Pal, Arpita Das Gupta, Asok K. Mallik

**Affiliations:** ^1^Department of Chemistry, Acharya Jagadish Chandra Bose College, Kolkata 700 020, West Bengal, India; ^2^Department of Chemistry, Jadavpur University, Kolkata 700 032, West Bengal, India

## Abstract

Iodine-catalyzed reaction of indoles with *α*,*α*′-bis(arylmethylene)cyclopentanones afforded one diastereomer of the corresponding Michael adducts, namely, *E*-2-(3-indolylphenylmethyl)-5-phenylmethylenecyclopentanones, in a good yield. The products form a new group of indole derivatives.

## 1. Introduction

**Scheme 1:**
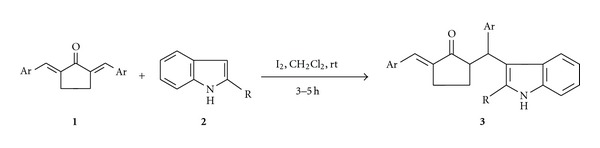
Iodine-catalyzed Michael addition of indoles to *α*,*α*′-bis(arylmethylene) cyclopentanones.

The Michael reaction has attracted much attention as one of the most important C–C bond-forming reactions [1, 2]. Indole is a very good Michael donor through its 3-position, and the 3-indolyl system is one of the important building blocks for many biologically active compounds, both natural and unnatural [3]. Traditionally, the Michael addition of indole to *α*,*β*-unsaturated ketones as well as addition reactions of indole to other electron deficient olefins is known to be catalyzed by strong bases and Bronsted acids [4–8]. In recent years, it has been reported that these reactions can also be promoted by Lewis acids [9–20]. Following a recent report of iodine-catalyzed Michael addition of indole to some *α*,*β*-unsaturated ketones in ethanol [13], we studied the reaction between indole and *α*,*α*′-bis(arylmethylene)cyclohexanones under the same condition when we got bis(3-indolyl)methylarenes instead of any conjugate addition product [21]. A survey of the literature revealed that the Michael reaction between indoles and *α*,*α*′-bis(arylmethylene)cyclopentanones has not been studied so far. We, therefore, carried out this work under the iodine-catalyzed condition and obtained Michael adducts in good yields (Scheme 1). The results of our study are presented herein.

## 2. Results and Discussion

 In a preliminary study, *α*,*α*′-bis(phenylmethylene)cyclopentanone** (1a)** was treated with indole** (2a)** (mole ratio 1 : 1) in the presence of 10 mol% iodine as catalyst in dry dichloromethane at room temperature. The reaction was found to be complete within 4 h affording the conjugate addition product (**3a)** in 60% yield. The reaction using double the amount of indole also gave the same product in a comparable yield (62%). Encouraged by this result, we carried out the reaction by a variation of conditions, the results of which are given in Table 1.

It is evident from the results presented in Table 1 that the reaction gave the best yield of the product when dichloromethane was used as solvent and catalyst iodine was used to the extent of 10 mol% (Table 1, Entry 1). When ethanol was used as solvent, the solubility of the substrates at room temperature as well as at the reflux temperature was a problem. 

**Figure 1:**
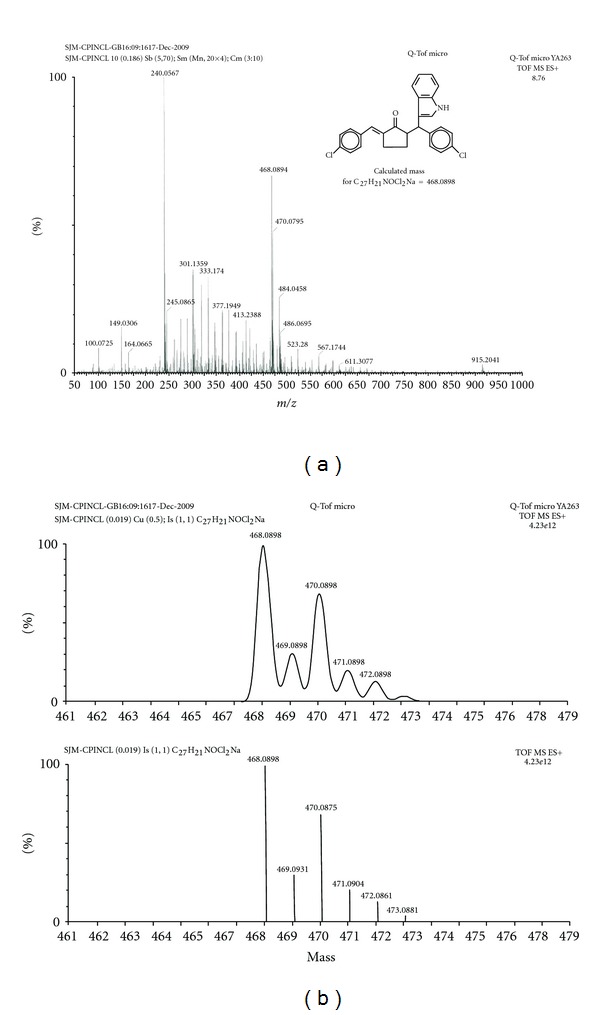
HRMS of compound **3c**.

Under the optimized reaction condition, the generality of the process was investigated with a number of *α*,*α*′-bis(arylmethylene)cyclopentanones (**1**), and the results are summarized in Table 2. It was noted that **1** with an electron withdrawing or a weakly electron donating group in the phenyl ring underwent facile reaction at room temperature affording the corresponding product within 3-4 h, while **1** with an electron donating group in the phenyl ring failed to undergo any reaction (Table 2, Entries 8 and 9) even at reflux temperature. *α*,*α*′-Bis(furfurylidene)cyclopetanone behaved like the latter group of substrates (Table 2, entry 10). Analytical and spectral data of the products definitely showed that they were *E*-2-(3-indolyl)-2-arylmethyl-5-arylmethylenecyclopentanones (**3**) (HRMS of 3c is shown in Figure 1). Another interesting point was that no bis(3-indolyl)methylarene formation was observed in this case. The reaction of **1** with one equivalent of 2-methylindole was then studied under similar conditions, which showed that this indole also reacted smoothly giving the corresponding products in a good yield (Table 2, Entries 5–7). Iodine-catalyzed reaction of *α*,*α*′-bis(arylmethylene)cyclohexanones with indole/2-methylindole in methylene chloride studied in this connection, however, gave bis(3-indolyl)methylarenes, just like that observed by the use of ethanol as solvent [21].

The Michael addition products **3** contain two adjacent stereogenic centres. It was an interesting observation that out of their two diastereomeric *dl*-pairs only one was formed. However, the configuration of the diastereomer formed could not be ascertained as none of **3a–g** formed good quality crystals from common organic solvents. *E*-Configuration has been suggested for them by considering that one of the phenylmethylene units of **1** remains completely unchanged during the reaction.

## 3. Conclusions

We have developed a simple and efficient method for Michael addition of indoles to *α*,*α*'-bis(arylmethylene)cyclopentanones using iodine as catalyst. The method is clean, and the products were obtained in a good yield without the formation of any side product. The Michael addition products **3a–g** are new compounds and may have potential biological activities.

## 4. Experimental Section

### 4.1. General

^1^H and ^13^C NMR spectra were obtained with a Bruker AV-300 (300 MHz) spectrometer in CDCl_3_ with TMS as an internal standard. IR spectra were recorded with a Perkin Elmer FT-IR Spectrophotometer (Spectrum BX II) as KBr pellets. Mass Spectra were recorded on Micro Mass Q TOF Spectrometer. Analytical samples were routinely dried invacuo at room temperature. Microanalytical data were recorded on two Perkin-Elmer 2400 Series II C, H, N analyzers. Column chromatography was performed on silica gel (100–200 mesh) using petroleum ether (60–80°C) and petroleum ether-ethyl acetate mixtures as eluents. TLC was done with silica gel G. *α*,*α*'-Bis(arylmethylene)-cycloalkanones were prepared by previously described methods [22, 23].

### 4.2. General Procedure

A mixture of an appropriate *α*,*α*′-bis(arylmethylene)cyclopentanone (**1**, 1 mmol) and an indole (1.1 mmol) was dissolved in dry dichloromethane (5 mL) at room temperature, and to the solution under stirring condition iodine (0.025 g, 10 mol%) was added. When the starting materials disappeared almost completely (after 3–5 h, checked by TLC), water (20 mL) was added to the reaction mixture, and it was extracted with dichloromethane (2 × 20 mL). The combined methylene chloride extract was washed with aqueous sodium thiosulphate solution (2 × 25 mL) and then dried over anhydrous sodium sulphate. The concentrate of the dichloromethane extract was subjected to column chromatography over silica gel (100–200 mesh) using a petroleum ether-ethyl acetate (9 : 1) as an eluent to obtain pure product, which was finally crystallized from chloroform-petroleum ether.

### 4.3. Physical and Spectral Data of E-2-(3-Indolylphenylmethyl)-5-phenylmethylene-cyclopentanones (**3a–g**)

#### 4.3.1. E-2-(3-Indolylphenylmethyl)-5-phenylmethylenecyclopentanone (**3a**)

(Entry 1 in Table 2): White solid; IR (KBr): 3490 (N–H), 2920 (ArC–H), 1701 (C=O), 1625, 1605 (C=C), 1495, 1420, 1240, 1160 cm^−1^; ^1^H NMR (300 MHz, CDCl_3_): *δ* 8.07 (br. s, 1H, N–H), 6.93–7.49 (m, 16H, Ar–H and C=C–H), 5.05 (d, 1H, *J* = 3.6 Hz, H-*β*), 3.24–3.29 (m, 1H, H-2), 2.69–2.83 (m, 2H, H_2_-4), 2.27–2.30 (m, 1H, H-3), 1.81–1.89 (m, 1H, H-3); ^13^C NMR (75 MHz, CDCl_3_): *δ* 207.54 (C=O), 141.67, 136.47, 136.27, 135.58, 132.67, 130.52, 130.39, 129.26, 128.62, 128.06, 129.91, 127.19, 126.34, 122.10, 121.59, 119.89, 119.31, 118.50, 110.92, 52.92 (C-*β*), 42.06 (C-2), 27.55 (C-4), 24.11 (C-3); Anal. Calcd for C_27_H_23_NO: C, 85.91; H, 6.14; N, 3.71; Found C, 85.68; H, 6.21; N, 3.49.

#### 4.3.2. E-2-(3-Indolyl-p-methylphenylmethyl)-5-p-methylphenylmethylenecyclopentanone (**3b**)

 (Entry 2 in Table 2): White solid; IR (KBr): 3380 (N–H), 3000 (ArC–H), 1698 (C=O), 1608, 1590 (C=C), 1496, 1426, 1238, 1165 cm^−1^; ^1^H NMR (300 MHz, CDCl_3_): *δ* 8.06 (br. s, 1H, N–H), 6.93–7.40 (m, 14H, Ar–H and C=C–H), 5.02 (d, 1H, *J* = 3.6 Hz, H-*β*,), 3.18–3.22 (m, 1H, H-2), 2.70–2.73 (m, 2H, H_2_-4), 2.37 (s, 3H, CH_3_), 2.22–2.31 (m, 4H, H-3 and CH_3_), 1.81–1.89 (m, 1H, H-3); ^13^C NMR (75 MHz, CDCl_3_): *δ* 207.01 (C=O), 140.50, 136.42, 135.63, 135.33, 132.64, 130.54, 129.35, 129.09, 128.88, 128.70, 128.09, 127.19, 122.93, 121.51, 119.88, 119.18, 117.13, 110.8, 52.98 (C-*β*), 41.82 (C-2), 27.42 (C-4), 24.42 (C-3), 21.38 (CH_3_), 20.93 (CH_3_); Anal. Calcd for C_29_H_27_NO: C, 85.89; H, 6.71; N, 3.45; Found C, 85.71; H, 6.53; N, 3.41.

#### 4.3.3. E-2-(3-Indolyl-p-chlorophenylmethyl)-5-p-chlorophenylmethylenecyclopentanone (**3c**) 

(Entry 3 in Table 2): White solid; IR (KBr): 3495 (N–H), 2910 (ArC–H), 1707 (C=O), 1620, 1600 (C=C), 1495, 1460, 1420, 1240, 1160, 1100 cm^−1^; ^1^H NMR (300 MHz, CDCl_3_): *δ* 8.11 (br. s, 1H, N–H), 6.95–7.42 (m, 14H, Ar–H and C=C–H), 5.06 (d, 1H, *J* = 3.6 Hz, H-*β*), 3.19–3.25 (m, 1H, H-2), 2.70–2.79 (m, 2H, H_2_-4), 2.30–2.35 (m, 1H, H-3), 1.75–1.83 (m, 1H, H-3); ^13^C NMR (75 MHz, CDCl_3_): *δ* 206.91 (C=O), 140.07, 136.46, 136.34, 135.31, 133.88, 132.14, 131.62, 131.57, 130.64, 128.9, 128.20, 126.91, 122.30, 121.53, 119.72, 119.45, 117.89, 110.98, 52.76 (C-*β*), 41.19 (C-2), 27.45 (C-4), 23.79 (C-3); HRMS observed for (M + Na)^+^ at 468.0894. Calculated for C_27_H_21_NOCl_2_ [M + Na]^+^ at 468.0898.

#### 4.3.4. E-2-(3-Indolyl-p-bromophenylmethyl)-5-p-bromophenylmethylenecyclopentanone (**3d**) 

(Entry 4 in Table 2): White solid; IR (KBr): 3371 (N–H), 2955 (ArC–H), 1692 (C=O), 1611, 1583 (C=C), 1487, 1404, 1290, 1152, 1073 cm^−1^; ^1^H NMR (300 MHz, CDCl_3_): *δ* 8.08 (br. s, 1H, N–H), 6.94–7.53 (m, 14H, Ar–H and C=C–H), 5.05 (d, 1H, *J* = 3.6 Hz, H-*β*), 3.19–3.27 (m, 1H, H-2), 2.69–2.77 (m, 2H, H_2_-4), 2.30–2.37 (m, 1H, H-3), 1.57–1.82 (m, 1H, H-3); ^13^C NMR (75 MHz, CDCl_3_): *δ* 206.88 (C=O), 140.63, 136.50, 134.33, 131.93, 131.87, 131.67, 131.2, 131.09, 126.93, 123.72, 122.36, 121.57, 120.34, 119.75, 119.52, 117.86111.02, 52.86 (C-*β*), 41.26 (C-2), 27.36 (C-4), 23.83 (C-3); Anal. Calcd for C_27_H_21_NOBr_2_ (535.27): C, 60.58; H, 3.95; N, 2.62; Found C, 60.43; H, 3.77; N, 2.85.

#### 4.3.5. E-2-[3-(2-Methyl)indolyl-phenylmethyl]-5-phenylmethylenecyclopentanone (**3e**) 

(Entry 5 in Table 2):White solid; IR (KBr): 3401 (N–H), 3024 (ArC–H), 2952, 1705 (C=O), 1622 (C=C), 1459, 1448, 1302, 1187, 741 cm^−1^; ^1^H NMR (300 MHz, CDCl_3_): *δ* 7.75 (br. s, 1H, N–H), 6.91–7.62 (m, 15H, Ar–H and C=C–H), 4.85 (d, 1H, *J* = 6.0 Hz, H-*β*), 3.52–3.55 (m, 1H, H-2), 2.73–2.78 (m, 2H, H_2_-4), 2.43 (s, 3H, Me), 2.31–2.39 (m, 1H, H-3), 1.85–1.90 (m, 1H, H-3); ^13^C NMR (75 MHz, CDCl_3_): *δ* 205.36 (C=O), 140.46, 135.28, 134.90, 132.65, 128.65, 128.49, 127.80, 120.61, 120.39, 118.95, 112.72, 109.95, 53.19 (C-*β*), 39.86 (C-2), 25.64 (C-4), 20.90 (C-3), 12.48 (Me); Anal. Calcd for C_28_H_25_NO (391.50): C, 85.90; H, 6.44; N, 3.58; Found C, 85.68; H, 6.50; N, 3.49.

#### 4.3.6. E-2-[3-(2-Methyl)indolyl-p-methylphenylmethyl]-5-p-methylphenylmethylenecyclopentanone (**3f**) 

(Entry 6 in Table 2): White solid; yield; IR (KBr): 3410 (N–H), 3022 (ArC–H), 2940, 1715 (C=O), 1612 (C=C), 1495, 1430, 1240, 1012, 750 cm^−1^; ^1^H NMR (300 MHz, CDCl_3_): *δ* 7.74 (br. s, 1H, N–H), 6.82–7.25 (m, 13H, Ar–H and C=C–H), 4.88 (d, 1H, *J* = 4.2 Hz, H-*β*), 2.78–2.82 (m, 1H, H-2), 2.35 (s, 3H, Me), 2.27–2.31 (m, 2H, H_2_-4), 2.25 (s, 6H, 2Me), 2.13–2.19 (m, 1H, H-3), 1.66–1.70 (m, 1H, H-3); Anal. Calcd for C_30_H_29_NO (419.56): C, 85.88; H, 6.97; N, 3.34; Found C, 86.01; H, 7.11; N, 3.29.

#### 4.3.7. E-2-[3-(2-Methyl)indolyl-p-chlorophenylmethyl]-5-p-chlorophenylmethylenecyclopentanone (**3g**)

 (Entry 7 in Table 2)*: *White solid; IR (KBr): 3396 (N–H), 3052 (ArC–H), 2953, 1706 (C=O), 1623 (C=C), 1490, 1459, 1237, 1091, 742 cm^−1^; ^1^H NMR (300 MHz, CDCl_3_): *δ* 7.78 (br. s, 1H, N–H), 7.17–7.40 (m, 11H, Ar–H and C=C–H), 7.65 (t, 1H, *J* = 6.9 Hz,), 6.94 (t, 1H, *J* = 7.8 Hz,), 4.74 (d, 1H, *J* = 6.6 Hz, H-*β*), 3.47–3.51 (m, 1H, H-2), 2.42 (s, 3H, Me), 2.70–2.75 (m, 2H, H_2_-4), 2.26–2.30 (m, 1H, H-3), 1.81–1.85 (m, 1H, H-3); Anal. Calcd for C_28_H_23_NCl_2_O (460.39): C, 73.05; H, 5.04; N, 3.04; Found C, 73.22; H, 4.90; N, 3.17.

## Figures and Tables

**Table 1:** Screening for optimum reaction conditions^a^.

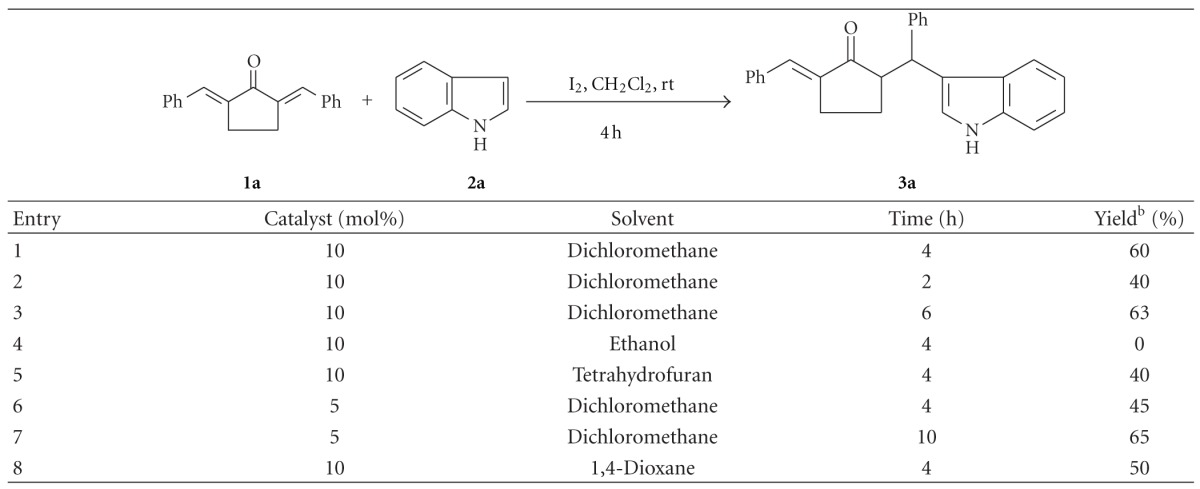

^a^Conditions: **1a** (1 mmol), **2a** (1.1 mmol), and solvent (5 mL).

^b^Yield after column chromatography.

**Table 2:** Results of Michael addition of indoles to *α*,*α*′-bis(arylmethylene)cyclopentanones catalyzed by iodine.

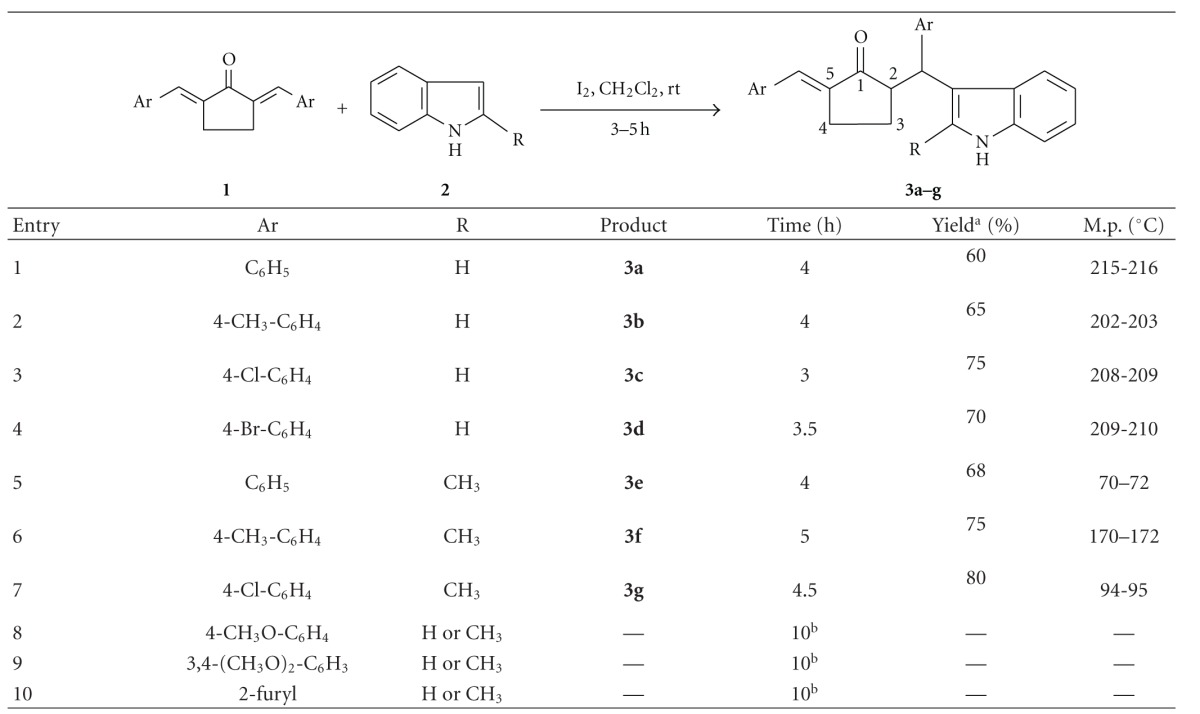

^a^Yield after column chromatography.

^b^The resulting crude material obtained after this time was mainly the starting compounds.
